# Assessing agreement between malaria slide density readings

**DOI:** 10.1186/1475-2875-9-4

**Published:** 2010-01-04

**Authors:** Neal Alexander, David Schellenberg, Billy Ngasala, Max Petzold, Chris Drakeley, Colin Sutherland

**Affiliations:** 1 Tropical Epidemiology Group, Department of Epidemiology and Public Health, London School of Hygiene and Tropical Medicine, London, UK; 2 Department of Microbiology, Immunology and Tropical Medicine, The George Washington University, Washington, USA; 3 Department of Infectious and Tropical Diseases, London School of Hygiene and Tropical Medicine, London, UK; 4 Infectious Diseases Unit, Department of Medicine, Karolinska University Hospital, Karolinska Institutet, Stockholm, Sweden; 5 Department of Parasitology, Muhimbili University of Health and Allied Sciences, Dar es Salaam, Tanzania; 6 Division of International Health, Karolinska Institutet, Stockholm, Sweden; 7 The Nordic school of Public Health, Gothenburg, Sweden; 8 Health Protection Agency Malaria Reference Laboratory, London School of Hygiene and Tropical Medicine, Keppel Street, London, UK

## Abstract

**Background:**

Several criteria have been used to assess agreement between replicate slide readings of malaria parasite density. Such criteria may be based on percent difference, or absolute difference, or a combination. Neither the rationale for choosing between these types of criteria, nor that for choosing the magnitude of difference which defines acceptable agreement, are clear. The current paper seeks a procedure which avoids the disadvantages of these current options and whose parameter values are more clearly justified.

**Methods and Results:**

Variation of parasite density within a slide is expected, even when it has been prepared from a homogeneous sample. This places lower limits on sensitivity and observer agreement, quantified by the Poisson distribution. This means that, if a criterion of fixed percent difference criterion is used for satisfactory agreement, the number of discrepant readings is over-estimated at low parasite densities. With a criterion of fixed absolute difference, the same happens at high parasite densities. For an ideal slide, following the Poisson distribution, a criterion based on a constant difference in square root counts would apply for all densities. This can be back-transformed to a difference in absolute counts, which, as expected, gives a wider range of acceptable agreement at higher average densities. In an example dataset from Tanzania, observed differences in square root counts correspond to a 95% limits of agreement of -2,800 and +2,500 parasites/μl at average density of 2,000 parasites/μl, and -6,200 and +5,700 parasites/μl at 10,000 parasites/μl. However, there were more outliers beyond those ranges at higher densities, meaning that actual coverage of these ranges was not a constant 95%, but decreased with density. In a second study, a trial of microscopist training, the corresponding ranges of agreement are wider and asymmetrical: -8,600 to +5,200/μl, and -19,200 to +11,700/μl, respectively. By comparison, the optimal limits of agreement, corresponding to Poisson variation, are ± 780 and ± 1,800 parasites/μl, respectively. The focus of this approach on the volume of blood read leads to other conclusions. For example, no matter how large a volume of blood is read, some densities are too low to be reliably detected, which in turn means that disagreements on slide positivity may simply result from within-slide variation, rather than reading errors.

**Conclusions:**

The proposed method defines limits of acceptable agreement in a way which allows for the natural increase in variability with parasite density. This includes defining the levels of between-reader variability, which are consistent with random variation: disagreements within these limits should not trigger additional readings. This approach merits investigation in other settings, in order to determine both the extent of its applicability, and appropriate numerical values for limits of agreement.

## Background

Blood slides remain a mainstay of malaria diagnosis for health services and research studies, including vaccine trials [1-3]. Despite the importance of quality assurance of slide readings [4-6], there are large differences between studies in terms of the amount of blood analysed[7,8] and the procedures for identifying and resolving discrepant results[4,9-11]. Even studies which do report their specific procedures rarely explain their rationale.

Parasite density is clinically important[12], with hyperparasitaemia being a characteristic of severe malaria[13]. In many studies, a proportion of parasite density estimates are checked by reading slides a second time. Various criteria have been used for satisfactory agreement between such paired readings. Some studies use a percent difference, for example 10%[10] or 50%[11]. One problem with this is that, at low densities, the low numbers of parasites seen may vary by large percentages, even in the absence of reading errors. For example, counts of 1 and 2 parasites (in a volume of, say, 100 microscopic fields) have a large percentage difference, but are perfectly consistent with a density of, for example, 1.5 per 100 fields. So it would not be reasonable to find them discrepant. Therefore, a fixed percentage threshold does not seem a suitable criterion for defining satisfactory agreement. On the other hand, a fixed difference in absolute counts does not seem suitable either because the risk of clinical manifestations, in particular fever, increases non-linearly with parasite density [12]. This means that, for example, the difference between 1 and 101 parasites is likely to be a greater concern than that between 1,001 and 1,101. In other words, neither a constant percent difference nor a constant absolute difference is a suitable criterion for defining satisfactory agreement.

Such considerations presumably led Alonso *et al *[9] and Bejon *et al *[14] to use criteria for satisfactory agreement which depend on the total number of parasites seen in two readings of the same slide. In the former study, a difference criterion (no more than 10 parasites) was used if fewer than 30 parasites were seen in total, otherwise a fold difference of 1.5. The latter study used a criterion of tenfold difference when one or both densities were below 400 parasites/μl, otherwise a twofold difference. However, no rationale is given for these threshold values.

Several types of rationale could be used to define discrepant pairs of readings. They could be defined as: 1) those whose differences lie outside a previously established typical range, 2) those whose differences are like to have clinical importance, or 3) those which exceed the minimum variation expected on theoretical grounds. As noted above, published studies generally state no rationale for the procedures used. The current paper concentrates on the first and third rationales, although clinical importance is considered briefly in the discussion.

On this basis, a framework is proposed for: a) assessing agreement between repeat readings, b) deciding an appropriate volume of blood to read, and c) how to reach a consensus result. These are important components of a broader quality control scheme, which, in turn, should be part of a quality assurance process. The approach will be illustrated by analysis of two datasets from Tanzania.

## Methods and results

### Repeatability and limits of agreement

Repeatability is a parameter which quantifies degree of agreement between multiple measurements of the same quantity of interest; in this case, parasite density. Unlike some measures of agreement, such as kappa, repeatability has the same units as the original quantity. A precise definition of repeatability is given by Braun-Munzinger and Southgate[15], following British Standard 5497:

Repeatability (*r*) is the value below which the absolute difference between two single test results obtained under repeatability conditions may be expected to lie with a probability of 95%.

Repeatability conditions are conditions where mutually independent test results are obtained with the same method in identical test material in the same laboratory by the same operator using the same equipment within short intervals of time.

The current paper will use a similar, but slightly looser, definition, effectively ignoring the above 'repeatability conditions', so that repeatability refers to the 95% range of differences between readings, irrespective of laboratory location, and whether done by the same or different readers.

Repeatability is related to Bland and Altman's 'limits of agreement'[16], which can be used to identify potentially discrepant readings. When the between-reading differences are normally distributed, then the limits of agreement are defined as the mean difference, plus and minus 1.96 standard deviations of the differences. These limits will, therefore, enclose approximately 95% of the data. Alternatively, as done in the current paper, the limits of agreement can simply be defined in terms of the actual 95% range of the data, hence avoiding the need to assume a normal distribution. This 95% range could be defined by the 2.5^th ^and 97.5^th ^percentiles, as done in the current paper. Alternatively, if the differences are symmetrical about zero, the 95^th ^percentile of the absolute values of the differences could be calculated, with plus and minus this percentile being used as the range limits. When the distribution of the differences is symmetric about zero, the repeatability (*r*) is half the distance between the upper and lower limits of agreement.

This procedure depends on the data having constant variability over the data range. If the variability is not constant, for example if it increases with magnitude, as expected with slide readings, then a value calculated from one dataset will not be applicable to another dataset with a different profile of parasite densities. For data whose variation increases with absolute value, it may be possible to calculate the limits of agreement after a logarithmic transformation. However, for parasite counts, this is likely to lead to the reverse problem of the variability decreasing with density [17-19]. Moreover, zero counts are difficult to accommodate, because they become infinite when log-transformed.

The concepts of repeatability and limits of agreement are, in principle, suitable for quantitative data, such as parasite counts, whereas some other measures of agreement, such as kappa (*k*), are more applicable for categorical data. Repeatability, or limits of agreement, can be used to define a threshold of satisfactory agreement. For example, two readings could be considered discrepant if they differ by more than the repeatability, i.e. if their difference is outside the 95% range of the differences of previous paired readings, i.e. outside the limits of agreement.

To apply such a method, it is necessary to define the specific quantity whose repeatability will be calculated. But, as seen above, neither the absolute difference in counts, nor the log-transformed counts, is likely to be satisfactory. Recent work on hookworm egg counts[20] suggests that the differences in square root counts may be a suitable quantity since, on this scale, the between-reading variation is constant over the data range (for the datasets examined). The difference in square roots compresses variation at higher densities, but not as strongly as the logarithmic transformation. So, rather than using a difference criterion of agreement at lower densities, and a ratio criterion at higher ones[9], the aim here is to investigate the use a constant difference in square roots throughout the data range.

Analysis of transformed data often has the disadvantage of losing the original scale of measurement. It is sometimes said that the logarithmic transformation is the only one to avoid have this disadvantage, because it is the only one whose results can be back-transformed to the original scale [21]. Repeatability of square roots is an exception because, if it is constant over the data range, then it too can be back-transformed to the original scale[20]. More specifically, if the variation is constant on the square root scale, and both readings are made from the same volume, then the repeatability of the differences in (untransformed) counts is proportional to the square root of the square mean root (SMR) count. The SMR is the square of the average of the square roots. If the two counts are *x*_1 _and *x*_2 _then the square mean root count is . This is a measure of location, which lies between the arithmetic and geometric means. In fact, when there are only two data points, the square mean root lies exactly halfway between those other two types of mean.

If the between-reader variability, as measured by the repeatability (*r*), is constant then, in the Bland & Altman plot of the difference in square roots versus their average, the limits of agreement are defined by lines with the following equations:

where *x*_1 _and *x*_2 _are the two parasite counts. Multiplying both sides by  gives:(1)

So the back-transformation converts horizontal lines to ones which have slope 2*r *when the difference in untransformed counts is plotted against the square root of the SMR. This assumes the limits of agreement are symmetric about the mean, i.e. that the mean difference is zero, but the same procedure is applicable to any line which is horizontal on the square root scale.

### A lower bound on repeatability

Even if two observers read slides perfectly, they will not generally count exactly the same number of parasites from the same slide (unless they read exactly the same section of it). Even a homogeneous blood sample will yield a slide with some variation between sections. This minimum variation is described by the Poisson distribution[22] which is applied to the actual number of parasites seen, before any transformation to a standard volume (eg per microliter). It so happens that the square root is the transformation which 'stabilizes the variance' of the Poisson, i.e. results in approximately constant variance over the data range[23]. This is an explanation for the square root transformation having this variance-stabilizing property, at least in some datasets. Moreover, it allows us to put a lower limit on repeatability. The variance of the square root of a Poisson variate is approximately 0.25[23]. So, a difference in two square roots of two Poisson variates is 0.5, and the standard deviation is 1/. Under a Normal approximation for the distribution in the difference in square roots, the limits of agreement would be ± 1.96/, or ± 1.39.

It is well known that parasite counts generally have between-person variation which is greater than Poisson ('overdispersion')[24]. However, the current analysis compares counts within each patient -- in fact, within a single slide -- so the variation is expected to be less. Apart from lack of independence between the paired readings (e.g. due to overlap between the sections of the slide which were read, or collusion between the readers), any deviation from this ideal situation of Poisson variation would be expected to increase repeatability (i.e. the between-reader variation). Such factors could include:

heterogeneous distribution of parasites on the slide, e.g. more dense towards the edges of a film[25];

reading errors, i.e. false positive or false negative identification of parasites;

differences between the readers in terms of the volume of blood read which may result from, for example, the thickness of the blood film varying over the slide [26];

conversion of parasite densities from per white cell to per microliter on the basis of a white cell count which is assumed constant but actually varies between people [27], or which seems less on the slide than an individual's externally calculated value, due to staining losses [26]. In fact, it may be more accurate to count a certain number of fields rather than adjusting for white cell count [28].

If one or more of these factors is present, the lower bound on repeatability is still important. This is because, if the agreement between a pair of readings is within the corresponding limits of agreement (i.e. their difference in square roots is between -1.39 and +1.39), then it is futile to declare the readings discrepant, even if published criteria indicate otherwise.

The methods described here can be implemented using the spreadsheet provided as Additional file 1.

### Example datasets

The method is illustrated with two datasests (Table 1). The first contains repeat readings of asexual parasites taken from cross-sectional surveys of malaria in north-eastern Tanzania [29]. Giemsa-stained blood films were examined by oil-immersion microscopy. One hundred fields were screened before a slide was deemed to be negative; if parasites were observed, they were counted against 200 white blood cells (WBCs). Hence the volume read was not exactly the same for all slides. However this additional source of variation will be ignored because, as shown in the Appendix, the impact on the estimated density is a difference of at most one parasite. Ethical approval for the surveys was granted by the National Institute of Medical Research (Tanzania) and the London School of Hygiene and Tropical Medicine (UK). 1,601 thick films people aged 0-45 years were read by two different microscopists. The current analysis does not consider the 37 (2.3%) who were negative on both readings, leaving 1564 pairs. This is based on the rationale that a) the of such double-zero pairs is unproblematic and b) the variance-stabilization of the Poisson distribution does not apply if the mean is zero.

**Table 1:** Characteristics of the two studies and results of their analysis

First author of study	Drakeley[29]	Ngasala[30]
Objective of study	evaluation of associations between parasite prevalence, altitude and rainfall	evaluation of training in clinical and microscopical diagnosis
Location	two regions of north-eastern Tanzania	two coastal districts north of Dar es Salaam Tanzania,
Study design	population based cross-sectional surveys along altitude transects in those aged up to 45 years	cluster-randomized trial with slides taken from febrile children aged under five years presenting to primary health care (PHC) facilities
Total number of paired readings in dataset	1,601	973
Number of double-zero pairs excluded	37	345
Number of pairs excluded due to missing readings	0	39
Number of pairs excluded due to semi-quantitative readings	0	61
Numbers of paired readings analysed	1,564	528
Mean difference in square root counts (95% confidence interval, p value)	(not done because the dataset did not identify individual readers)	-1.51 (-2.1 to -0.95, p < 0.0001), with central laboratory tending to read higher than PHC
95% limits of agreement in terms of square root counts, i.e. 2.5 and 97.5 percentiles (ideal limits are -1.39 to +1.39)	-5.3 to +4.7	15.2 to +9.2
95% limits of agreements in parasites/μl at average density of 2,000 parasites/μl (ideal limits are ± 780 parasites/μl)	-2,800 to +2,500	8,600 to +5,200/μl
95% limits of agreements in terms of parasites/μl at average density of 10,000 parasites/μl (ideal limits are ± 1,800)	-6,200 to +5,700	-19,200 to +11,700

A second dataset is from a randomized controlled trial of training in clinical and microscopical diagnosis, also from Tanzania[30]. Slide readings methods were similar to the previous study. Ethical approval for the trial was granted by National Institute for Medical Research (Tanzania) and the Karolinska Institutet (Sweden, reference D-nr 03-712). The blood volumes read were the same as for the above study. The dataset contained 973 paired readings. Of these, the following were not included in further analysis: 345 (35%) in which both readings were zero; 39 (4%) with one reading missing; and 61 (6%) in which at least one reading was 1,000, 2,000 or 5,000, these apparently being semi-quantitative estimates rather than exact counts. Hence, 528 pairs were analysed.

**Figure 1:**
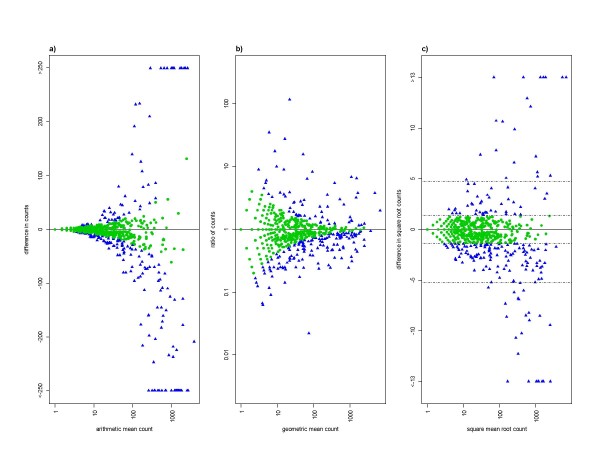
**Variation between readers in asexual parasites counts from cross-sectional surveys of malaria in northeastern Tanzania**[29]. The vertical axes show between-reader differences in terms of a) absolute difference in counts, b) ratio of counts and c) difference in square root counts. Different types of mean are used on the horizontal axes to ensure that the two axes are uncorrelated: arithmetic mean, geometric mean, and square mean root, respectively. Pairs with a zero reading (n = 702, or 45% of 1564) are not included in panel b) because they give rise to infinite ratios. To increase comparability, they also are omitted from the other two panels. Hence each panel shows 862 paired readings. In panel a), differences in either direction of more than 250 parasites (42 pairs, or 5% of 862) are plotted together at the ends of the vertical axis. This is to stop outliers dominating the figure. This is also done in panel c) for differences in square roots greater than 13 (16 pairs, or 2%). In panel c), the outer pair of horizontal lines shows the 95% range of the difference in square roots: -5.3 to 4.7. The inner pair of horizontal lines shows the range expected under the lower limit on this 95% range from the Poisson distribution: -1.39 to +1.39. In all three panels, the points are green circles or blue triangles, according to whether their difference in square roots is inside or outside this range.

Figure 1 shows the between-reader variation of the paired readings for the first dataset. This is shown in three ways: in terms of difference in counts, ratio of counts, and difference in square root counts. For differences in counts, the variation increases with intensity (Figure 1a); for ratios, it decreases with intensity (Figure 1a); and for differences in square roots the variation is roughly constant, although with some tendency to increase with intensity (Figure 1c). In terms of square root counts, the limits of agreement are calculated as the 2.5^th ^and 97.5^th ^percentiles of the differences in square roots, which are -5.3 and 4.7. These are the outer pair of horizontal dashed lines in Figure 1c. The inner pair of dashed lines shows the range expected under the lower limit on this 95% range from the Poisson distribution, i.e. -1.39 (= -1.96/v2) to +1.39.

Figure 1 also shows those paired readings whose differences lie in the 95% range expected under the Poisson distribution (i.e. with difference in square roots less than 1.39). Since this is an ideal minimum degree of variation, such differences are easily explained by random variation, and it would serve no purpose to send such slides to be read a third time. The figure shows, that, at high densities, large differences in absolute counts can arise by chance, as can large ratios at low densities. Therefore, a constant difference or ratio, if used as a definition of discrepant readings, will falsely identify some pairs of readings whose differences are likely to have arisen by chance. By contrast, applying a constant difference in square roots ensures that the proportion of chance differences which are falsely identified as discrepancies varies less with parasite density. In other words, in terms of identifying genuinely discrepant readings, a rule based on difference in square roots is preferable in terms of specificity.

**Figure 2:**
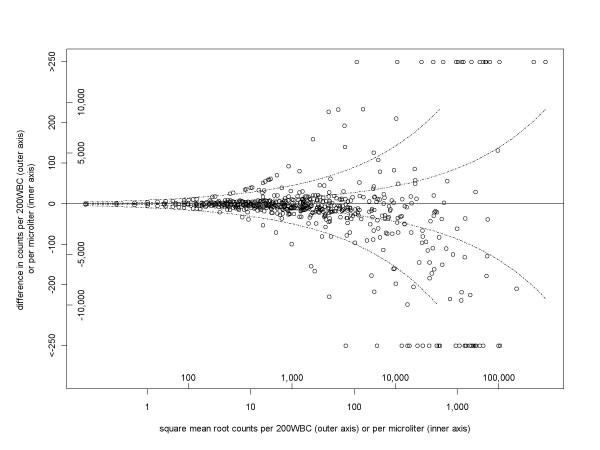
**Variation between readers increasing with parasite density: cross-section study**[29]. The vertical axis is the between-reader difference in terms of numbers actually counted (outer vertical axis) or in density per microliter (assuming 8,000 white blood cells per microliter, inner vertical axis). This is a back-transformation of Figure 1c, except that pairs with a zero reading are included, giving 1,564 pairs. The 95% range of difference in square roots is -4.9 to +4.5. These values are shown in the outer pair of curved dashed lines, corresponding to horizontal lines in Figure 1c. The inner pair of dashed lines is the lower limit on this 95% range, derived from the Poisson distribution (-1.39 to +1.39). As done for Figure 1c, differences of more than 250 are shown together at the limits of the vertical axis.

Figure 2 shows the back-transformation of Figure 1c, so that the vertical axis shows the difference in absolute counts. The two outer dashed lines correspond to the 95% range of the observed difference in square roots, and become further apart at higher densities. These can be used as 95% limits of agreement. At an average density of 2,000 parasites/μl they are -2,800 and +2,500 parasites/μl, and at 10,000 parasites/μl they are -6,200 and +5,700 parasites/μl. However, there were more outliers beyond those ranges at higher densities, meaning that actual coverage of these ranges was not a maintained constant at the nominal level of 95%, but decreased with density. The present analysis does not indicate whether this is because there are greater reading errors at higher densities or the sensitivity of the method is lower at lower densities. The inner pair of dashed lines in Figure 2 shows the 95% optimal limits of agreement, corresponding to Poisson variation. These are ± 780 and ± 1,800 parasites/μl, respectively, at average densities of 2,000 and 10,000 parasites/μl.

**Figure 3:**
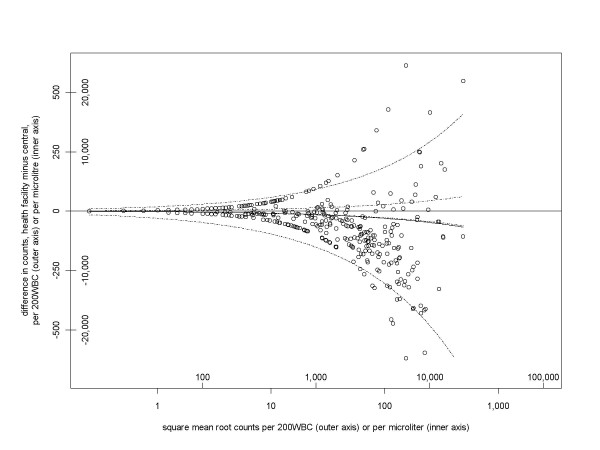
**Between-reader variation for trial of training methods**[30]. The axes are as in Figure 2. The 95% range of difference in square roots for the 528 pairs is -15.2 to +9.2, which transform to the outer pair of curved dashed lines. The inner pair of dashed lines is the lower limit on this 95% range (-1.39 to +1.39), derived from the Poisson distribution.

Figure 3 is similar to Figure 2, but for the trial of training methods[30]. The 95% range of the differences in square root counts is from -15.2 to +9.2. These have been transformed to the outer pair of dashed lines in Figure 3. At average densities and 2,000 and 10,000 parasites/μl, the corresponding 95% ranges of agreement are from -8,600 to +5,200/μl, and from -19,200 to +11,700/μl, respectively. As before, the actual coverage of the dashed lines decreases with greater parasite density. This dataset shows greater between-reader variation than the previous one, which seems likely to be due to the lesser experience and training of some of the readers.

It is also noticeable in that the readings in the central laboratory tended to be higher than those at the health facility. This is evident in the balance of points below and above the horizontal axis, and the resulting asymmetry in the outer pair of dashed lines. The mean difference in square root counts is -1.51. A *t *test gives a *p *value of less than 0.0001 (95% confidence interval for the mean difference in square roots is -2.1 to -0.95), indicating that this difference is unlikely to have occurred by chance. (Such an analysis was not done for the other dataset because it did not identify individual readers.) This mean difference of -1.51 back-transforms to the solid curved line in Figure 3 (just below the lower of the inner pair of dashed lines), and corresponds to larger differences in counts at larger densities.

### Reading larger volumes reduces the limits of agreement

The repeatability, or limits of agreement, in terms of differences in actual counts can be expressed in terms of a standard volume, eg microliter. The limits of agreement will be wider if a larger conversion factor is needed, i.e. if the original readings were based on a smaller volume of blood. Hence, interpretation of between-reader variation should take into account the volume read, although sometimes this is not reported [14] or even stored in the computerized data.

**Figure 4:**
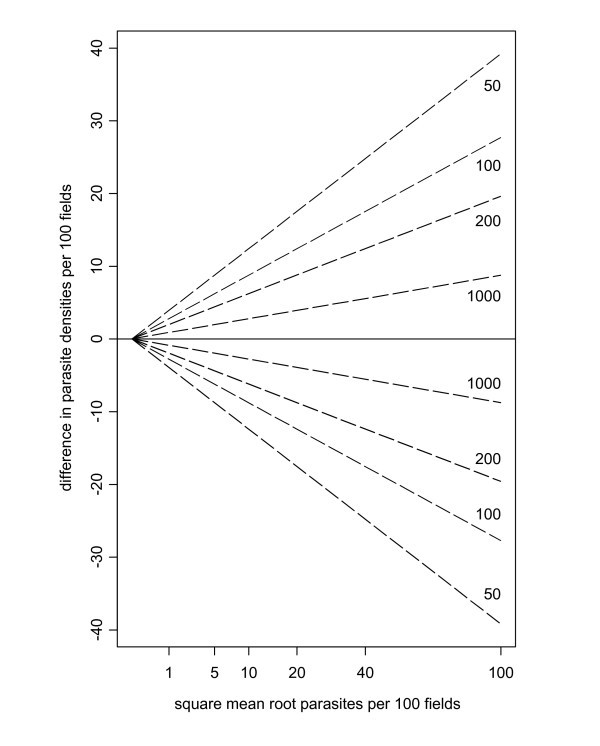
**Limits of agreement are wider when less blood is examined**. Ideal limits of agreement in terms of a standard volume of 100 fields. Each pair of dashed lines shows the limits of agreement for differing numbers of fields actually read, with the densities converted to the standard volume. The larger the volume read, the narrower the limits of agreement.

The specific relation between volume and repeatability can be obtained in terms of densities *y*_1 _and *y*_2 _per volume *V *by replacing *x *= (*v*/*V*)*y *in equation 1), where *v *is the volume read by each reader, yielding:(2)

where SMR_*y *_is the square mean root of *y*_1 _and *y*_2 _(i.e. the square of the mean of their square roots). In other words, for a fixed standard volume, the repeatability is inversely proportional to the square root of the volume read (i.e. there is greater variation for lower volumes read). This is illustrated in Figure 4.

### Distinguishing negative from positive slides

Some quality control procedures call for all slides with disagreements on infection status to be re-read[8,31]. Like most assays, slide reading has a lower detection limit. No matter how large a volume of blood is examined, the sensitivity will be low if the mean number of parasites in that volume is low. To be concrete, suppose that a slide is read twice, each time on 100 high power fields, and that one reading finds no parasites while the second reading finds one parasite. These two readings are completely consistent with a density of, for example, 0.5/100 hpf. If a third reading is done and found zero parasites, it would not necessarily be reasonable to discard the positive reading. It is worth remembering that with such a low-density the first two readings could easily have both been negative. In fact, the probability of disagreement between two error-free readers reaches 50% when the combination of parasite density and volume read is such that the latter contains zero parasites with a probability of 50%. For a homogeneous sample, under the Poisson distribution, this occurs when the volume read (*v*) is log_*e*_2 (≈0.693) divided by the density per unit volume (*l*).

If one of the readings finds no parasites, then the other should find at least 3 before being considered discrepant. This is because, if it is acceptable to falsely identify 5% of chance disagreements as discrepancies, √0 - √3 is greater than the Poisson-based limits of 1.39 (which is the 97.5% point of a normal distribution with mean zero and variance 1/2). Readings of 0 and 2 are also outside the range, but by a very small amount: 1.41 compared to 1.39, corresponding to a proportion of 4.9% rather than 5%. Hence it seems reasonable to set 3 as the lowest number of parasites to be considered discrepant with a paired count of 0, if 5% is taken as the cut-off proportion, following the definitions of repeatability[15] and limits of agreement[16]. If this proportion is, instead, set at 1%, then counts of 7 or more would be considered discrepant with a zero, because 7 is the smallest integer whose square root is larger 2.58, which is the 99.5% point of the normal distribution with mean zero and variance 1/2.

It is not possible to reliably detect infections of arbitrarily low density. A more realistic target is to specify the lowest density which one wants to reliably detect, and choose the volume of blood to be read accordingly. The minimum volume required can be derived from the ideal conditions of a homogeneous blood sample, and an absence of reading errors (so a parasite will be counted if present on the section of the slide which is read). Then, under the Poisson distribution, the probability of at least one parasite being seen is *p *= 1-*e*^-*lv*^, so *v *= -log_*e*_(1-*p*)/*l*. For example, to detect with 90% probability an infection of 1 parasite per 100 fields, the volume read should be 230 fields, or 460 fields to reach 99% probability. Allowance for false negatives and parasites being heterogeneously distributed over the slide would increase the volume required.

### Deriving a final reading when additional readings have been done

As well as defining the limits of acceptable agreement between readers, a quality control scheme should specify how to proceed when agreement is deemed unsatisfactory. For single pairs of readings, which are considered discrepant, according to the limits of agreement, a third reading could be made, with a consensus estimate of parasite density being obtained according to the following possible outcomes.

#### a) The third reading is in satisfactory agreement with only one of the original two

In this case, the two readings in satisfactory agreement (i.e. the third reading and one of the original two readings) would be used to calculate the final density.

#### b) The third reading is in satisfactory agreement with both of the original two

This is possible if the third reading lies between the first two. In this situation all three of the original slides, including the two originally judged to be discrepant, are used to calculate the final density.

#### c) The third reading is in satisfactory agreement with neither of the original two

This should be rare. However, should it happen, a fourth reading would be taken. The process would repeat till at least two readings are in satisfactory agreement, and the final reading would be based on those.

In any case, the process will end up with two or more readings from which the final estimate would be calculated. The final value would be the sum of parasites seen divided by the sum of areas (e.g. microscopic fields) counted and, if necessary, then converted to a standard volume. If all readings were done on the same volume, this corresponds to the arithmetic mean of the estimated densities. For the ideal situation of Poisson variation, this is the maximum likelihood estimate of the density. In practice there may be some justification for using a different kind of mean, such as the geometric mean[14], which effectively gives less weight to higher values. The geometric mean is zero when any of the individual readings is zero. This may be a disadvantage because, as argued above, a combination of zero and positive readings is to be expected at low enough densities, and does not necessarily reflect reading errors. In addition, it may be worth periodically comparing all the readings of each pair of readers. Wide limits of agreement (i.e. large repeatability) for particular readers may suggest systematic problems, which it may be possible to address, possibly through additional training. On the other hand, if some pairs of readers have unexpectedly narrow limits of agreement, perhaps less than the Poisson theoretical minimum, this would suggest that the readings are not mutually blind.

## Discussion

This method for assessing agreement of malaria slide densities may prove capable of replacing the various, somewhat arbitrary, definitions of acceptable agreement which have been used recently[4,9-11]. The utility of this framework will depend on its applicability to additional datasets, and this requires analysis of the unadjusted parasite counts, i.e. the numbers actually seen. Unfortunately, few studies seem to retain these in their computerized data files, only the densities after conversion to a standard unit volume. Agreement between readers cannot be fully assessed in terms of these standardized densities alone, because their variability depends on the volume of blood actually read. The method described here merits further evaluation, which would involve analysing the actual numbers of parasites seen, and evaluating whether the square root transformation stabilizes the variance across the range of densities. If so, values of repeatability and limits of agreement can be determined that would be rational definitions of acceptable agreement.

Algorithms which use a constant difference, or a constant ratio, between two readers' parasite counts as a criterion for triggering a third reading are bound to falsely identify a large proportion of chance differences as discrepancies, either at high or at low densities, respectively. This can be seen by deriving a lower bound on between-reader variation, based on the ideal distribution of parasites on a slide. Departures from this distribution -- other than readers colluding, or counting the same area of the slide -- would increase the between-reader variation above the ideal level.

The Poisson distribution can be used to describe parasites uniformly distributed on a blood slide or counting chamber[22]. Raghavan [32] recognized that infections may be missed simply due to random variation within a slide. However, the corresponding probabilities were estimated from the binomial distribution, by postulating a certain number of parasites in a certain number of fields. The problem is that if, for example, there are exactly 10 parasites in a certain set of 1,000 fields, the next set of 1,000 will not necessarily also contain 10. This variation across the sample can be described by the Poisson distribution. The corresponding results are similar to Raghavan's at low densities but diverge as density increases.

This Poisson lower bound on between-reader variation can be expressed in terms of a difference in square root parasite counts. Under ideal conditions, the square roots of paired readings will differ by more than 1.39 5% of the time, and by more than 2.58 1% of the time. If a pair of readings differ by an amount, which could have plausibly arisen by chance, then it is futile to declare them to be discrepant. If, in terms of density per microliter, such limits are considered unacceptably wide, e.g. on clinical grounds, then the solution should be to read a greater blood volume and hence reduce the extent of random variation (Figure 4).

What remains unclear is how far, if at all, the required repeatability should exceed this lower limit. It may be that, for example, a certain magnitude of between-reader difference may be in excess of chance but does not correspond to a clinically important difference, and hence does not merit a third reading. Such an approach would require an algorithm for determining which differences were clinically important. Smith *et al *[12] found that the log-odds of clinical disease did not increase linearly with the logarithm of parasite density, but was better explained by a power-law relationship. Future work could conceivably link such a relationship back to criteria for adequate agreement between readers. A second approach would be to define acceptable agreement in terms of average levels previously achieved. This comparison could be done against results established from previous work of the same laboratory (e.g. the outer pair of dashed lines in Figure 2). The agreement criteria could also be based on data from other laboratories, such as a reference centre whose accuracy could be considered as exemplary. This approach could form part of an external quality assurance scheme[33]. Since the criteria are based on 2.5 and 97.5 percentiles, the datasets should be reasonably large, with at least several hundred paired readings. The data analysed here show that different studies can very different limits of agreement, so ones with wider limits may benefit from being compared against a stricter standard from a different laboratory.

There is scope to develop the analytic framework presented here to situations where the variation in between-reader differences in square root counts increases with parasite density, as happens to some extent in the datasets analysed here. In particular, quantile regression[34] could be used to fit the 2.5 and 97.5% percentiles of the difference in square roots as linear functions of the average in square roots. Then, the difference in actual counts would be proportional to the the square mean root average count, rather than the square root of the average as above. However, some of the large differences are likely to have resulted from errors in reading, and it remains to be seen how much emphasis should go on making the analysis reflect such empirical variations, and how much on trying to reduce the between-reader variation as close as possible to the ideal Poisson situation, which would result in constant variability in square roots.

This paper has assumed that all the slide readings being compared have been done on the same volume of blood, although this is not always the case [35]. Some procedures call for an extended volume to be read if no parasite is seen in an initial volume[36]. Whatever the volume of blood read, however, there are some densities too low to be reliably detected. This constraint, which is sometimes recognised in the reporting of sensitivity according to density category[37], should be taken into account by authors who set targets for increased sensitivity of microscopy[38]. Similarly, studies which compare readers against a gold standard composed of one or more other readers[39] should recognize that some disagreements are inevitable, even for error-free readers, because of variations that exist across any blood film, including those made from homogeneous samples. This minimum level of disagreement between two error-free readers can be expected to reach 50% for some combinations of density and volume read. At low densities, other procedures may be appropriate, e.g. asking a colleague to verify whether an object currently visible is indeed a parasite.

Acceptable operating characteristics of microscopical diagnosis may differ between applications. For example, surveillance over a large area may need to detect low density infections with greater sensitivity than a Phase III trial in an area of high transmission. This in turn may dictate the possible role of alternative diagnostic methods, e.g. polymerase chain reaction (PCR).

The emphasis has been on the agreement of infection densities between slide readers, rather than on other types of error, such as disagreements on the *Plasmodium *species present. Moreover, slide reading itself cannot be considered in isolation, since its reliability depends on other factors, which should be considered as part of a comprehensive quality control and quality assurance process. These include: a suitable working environment, standard operating procedures, optimum slide preparation, continuing staff training, review of results in conjunction with a reference centre, equipment maintenance, power supply for microscopes, logistical systems to maintain supplies, and appropriate funding mechanisms[4,6,37,40].

## Conclusions

The proposed method defines limits of acceptable agreement, which follow the natural increase in variability with parasite density. A lower bound on between-reader variation can be obtained from the Poisson distribution: 95% of differences in square root counts between -1.39 and +1.39. Any disagreements which lie within these limits should not trigger additional readings. This stage of the analysis is done on numbers of parasites actually seen, rather than in terms of a standard volume such as microliter. However, the results can be back-transformed to differences in counts per unit volume, giving limits of agreement which increase with density, and are wider if the volume originally read was smaller. Disagreements on slide positivity may simply result from within-slide variation, rather than reading errors. Again, this variation may be reduced but not eliminated by increasing the volume of blood read. In two actual datasets, the between-reader variation was much greater than the Poisson lower bound, although was largely, but not completely, stabilized over the data range by the square root transformation. When back-transformed to the original scale, the limits of agreements increase with parasite density and are much wider for the study trained microscopists, compared to the other study. This approach merits investigation in other settings, in order to determine both the extent of its applicability, and appropriate numerical values for limits of agreement.

## Competing interests

The authors declare that they have no competing interests.

## Authors' contributions

NA devised the method for quantifying agreement. Its application to malaria slides was developed in conjunction with DS and CS. MP, BN and CD made available datasets and aided NA in analysis and interpretation. All authors read and approved the final manuscript.

## Appendix: Estimating parasite density from varying volumes of blood

In some laboratories, a certain volume of blood (say *u *μl) is read before deeming a slide to be negative but, if parasites are seen, then an additional volume (say *v *μl) is read. For example, *u *may correspond to 100 fields, or about 0.1-0.25 μl [41], while *v *may correspond to 200 white blood cells, or 0.025 μl, assuming 8,000 white blood cells per microliter. Under this procedure, the volume of blood read is not the same for all slides, and estimation of parasite density depends on the volumes *u *and *v*.

Assuming a homogeneous (Poisson) distribution of parasites on the slide, with density *λ*/μl, then the probability of seeing no parasites is e^-*λu*^, and of seeing a number *x *(>0) is (1- e^-*λu*^) e^-*λv *^(*λv*)^*x*-1^/(*x*-1)!. This second probability is the product of a) the probability of seeing a parasite before the end of the first volume *u*, and b) the Poisson probability of finding *x*-1 more parasites in the volume *v *(hence reaching the specified total *x*). Overall, the expected number of parasites seen is (1- e^-*λu*^)(*λv *+ 1). This is the expected number of parasites in volume *v*, plus the initial single parasite, times the probability of seeing at least one parasite.

For a single slide reading, the density *λ *can be estimated by maximum likelihood. For *x *= 0 the likelihood is e^-*λu *^which, as expected, is maximized by *λ *= 0. For positive *x*, the likelihood is (1- e^-*λu*^) e^-*λv *^(*λv*)^*x*-1^/(*x*-1)!. An explicit algebraic solution is not possible, but a numerical solution can be obtained by computer. For large values of *x*, the estimate of *λ *is very close to that obtained by dividing *x*-1 by *v*, i.e. as if only the volume *v *had been read, disregarding the single parasite in the initial volume *u*.

To be more specific it is necessary to assign values to *u *and *v*. The impact of the initial volume *u *is greater at smaller values, because for large values the term 1- e^-*λu *^is close to 1. Hence an illustrative choice is *u *= 0.1 μl, at the lower end of the range given above, with *v *being 0.025 μl. For these values, for *x *= 3 the solution is 80.1 parasites/μl, close to the 80/μl which would be obtained by dividing (*x*-1) by *v*. In other words, the estimated average density is 2.003 parasites per 200 WBC. For larger values of *x*, the solution is even closer to (*x*-1)/*v*. For smaller values, *x *= 2 gives an estimate of 42.5/μl, or 1.06/200 WBC. For *x *= 1 (i.e. no parasites seen in the second volume *v*), the estimate is 16.1/μl, or 0.40/200 WBC. Hence, overall, at most one parasite should be subtracted from the total number actually seen, before dividing by *v*.

## Supplementary Material

Additional file 1 **Malaria slide density agreement**. A spreadsheet which can be used to implement the method described int the paper. Blue cells, and columns headed in blue, are to be supplied by the user. Yellow cells, and columns headed in yellow, are calculated by the spreadsheet. The data (numbers of parasites actually seen, not per microlitre) should be pasted into columns 'reader 1' and 'reader 2'. It may be convenient to put an identifying variable in the 'ID' column. We suggest omitting double-zero counts from analysis (see main text for rationale). The 'ScratchData' tab is not intended to be modified by the user. However, if more than 1000 paired readings are needed, additional rows can be copied on to the bottom of the 'data' and 'ScratchData' tabs. The last four tabs contain different graphs of the data. Click here for file
